# Effectiveness of a community-based rehabilitation programme following hip fracture: results from the Fracture in the Elderly Multidisciplinary Rehabilitation phase III (FEMuR III) randomised controlled trial

**DOI:** 10.1136/bmjopen-2024-091603

**Published:** 2025-05-12

**Authors:** Nefyn Williams, Monica Busse, Rachael Cooper, Susanna Dodd, Shanaz Dorkenoo, Kodchawan Doungsong, Rhiannon Tudor Edwards, Jessica Green, Ben Hardwick, Andrew Lemmey, Phillipa Logan, Valerie Morrison, Penelope Ralph, Catherine Sackley, Benjamin Edward Smith, Toby Smith, Llinos Haf Spencer

**Affiliations:** 1 Department of Primary Care and Mental Health, University of Liverpool, Liverpool, UK; 2 Centre for Trials Research, Cardiff University, Cardiff, UK; 3 Liverpool Clinical Trials Centre, University of Liverpool, Liverpool, UK; 4 Data Science, University of Liverpool, Liverpool, UK; 5 Involving People, Cardiff, UK; 6 Centre for Health Economics and Medicines Evaluation, Bangor University, Bangor, UK; 7 School of Sports, Health and Exercise Science, Bangor University, Bangor, UK; 8 Community Health Sciences, The University of Nottingham, Nottingham, UK; 9 Bangor University, School of Psychology, Bangor, UK; 10 University of Nottingham, Nottingham, UK; 11 Physiotherapy Outpatients, Derby Teaching Hospitals NHS Foundation Trust, Derby, UK; 12 Division of Rehabilitation and Ageing, University of Nottingham School of Medicine, Nottingham, UK; 13 University of Warwick, Coventry, UK; 14 Welsh Institute for Health and Social Care, University of South Wales, Pontypridd, UK

**Keywords:** Randomized Controlled Trial, REHABILITATION MEDICINE, Fractures, Closed, Hip, Pragmatic Clinical Trial

## Abstract

**Objective:**

To determine whether an enhanced community rehabilitation intervention (the Fracture in the Elderly Multidisciplinary Rehabilitation (FEMuR) intervention) was more effective than usual National Health Service care, following surgical repair of hip fracture, in terms of the recovery of activities of daily living (ADLs).

**Design:**

Definitive, pragmatic, multisite, parallel-group, two-armed, superiority randomised controlled trial with 1:1 allocation ratio.

**Setting:**

Participant recruitment in 13 hospitals across England and Wales, with the FEMuR intervention delivered in the community.

**Participants:**

Patients aged over 60 years, with mental capacity, recovering from surgical treatment for hip fracture and living in their own home prior to fracture.

**Interventions:**

Usual rehabilitation care (control) was compared with usual rehabilitation care plus the FEMuR intervention, which comprised a patient-held workbook and goal-setting diary to improve self-efficacy, and six additional therapy sessions delivered in-person in the community, or remotely during COVID-19 restrictions (intervention), to increase the practice of exercise and ADL.

**Primary and secondary outcome measures:**

Primary outcome was the Nottingham Extended Activities of Daily Living (NEADL) scale at 12 months. Secondary outcomes included: Hospital Anxiety and Depression Scale, Falls Self-Efficacy-International scale, hip pain intensity, fear of falling, grip strength and Short Physical Performance Battery. Outcomes were collected by research assistants in participants’ homes, whenever possible, but had to be collected remotely during COVID-19 restrictions.

**Results:**

In total, 205 participants were randomised (n=104 experimental; n=101 control). Trial processes were adversely affected by the COVID-19 pandemic. There were 20 deaths, 34 withdrawals and three lost to follow-up. At 52 weeks, there was no significant difference in NEADL score between the FEMuR intervention and control groups. Joint modelling analysis testing for difference in longitudinal outcome adjusted for missing values also found no significant difference with a mean difference of 0.1 (95% CI −1.1, 1.3). There were no significant between-group differences in secondary outcomes. Sensitivity analyses, examining the impact of COVID-19 restrictions, produced similar results. A median of 4.5 extra rehabilitation sessions were delivered to the FEMuR intervention group, with a median of two sessions delivered in-person. Instrumental variable regression did not find any effect of the amount of rehabilitation on the main outcome. There were 53 unrelated serious adverse events (SAEs) including 11 deaths in the control group: 41 SAEs including nine deaths in the FEMuR intervention group.

**Conclusions:**

The FEMuR intervention was not more effective than usual rehabilitation care. The trial was severely impacted by COVID-19. Possible reasons for lack of effect included limited intervention fidelity (fewer sessions than planned and remote delivery), lack of usual levels of support from health professionals and families, and change in recovery beliefs and behaviours during the pandemic.

**Trial registration number:**

ISRCTN28376407.

**STRENGTHS AND LIMITATIONS OF THIS STUDY**

- This Fracture in the Elderly Multidisciplinary Rehabilitation phase III (FEMuR III) randomised controlled trial was developed from previous FEMuR research, which followed the Medical Research Council framework for codeveloping an enhanced rehabilitation intervention and testing the feasibility of trial methods.
- Recruitment and follow-up assessments were adversely affected by the COVID-19 pandemic.
- In-person delivery of rehabilitation was not possible during the COVID-19 lockdown, but rehabilitation delivery continued using remote methods.
- Intervention fidelity was affected by the COVID-19 pandemic, with the intervention group receiving fewer additional therapy sessions than planned.

## Background

 Hip fracture (proximal femoral) is a common, major health problem in old age, affecting 72 160 people in England, Wales and Northern Ireland in 2022.^1^ It is expected to increase as the population ages.^2^ Mortality is high, with 28% dying within the first 12 months postfracture.^3^ Survivors experience worse mobility, less independence, worse quality of life and higher rates of institutionalisation compared with age-matched controls.^4^ Hip fractures cost the UK National Health Service (NHS) more than £2 billion a year, with most costs occurring outside of the acute hospital setting.^5^

The National Institute for Health and Care Excellence (NICE) recommends the provision of a co-ordinated multidisciplinary hip fracture programme including early identification of individual goals for multidisciplinary rehabilitation to recover mobility and independence, and efforts to facilitate return to prefracture residence and long-term well-being.^6^ Research recommendations from NICE guidance include a randomised controlled trial (RCT) to test the effectiveness and cost-effectiveness of additional physiotherapy or occupational therapy after hip fracture.

We aimed to address this research priority, following the Medical Research Council (MRC) framework for developing and evaluating complex interventions.^7^ In the first phase, we codeveloped the FEMuR (Fracture in the Elderly Multidisciplinary Rehabilitation) intervention.^8^ This was designed to enhance usual rehabilitation care in the community using a patient-held workbook and goal-setting diary, alongside six additional therapy sessions delivered in patients’ homes by physiotherapists or occupational therapists (logic model published previously^9^). The second phase was a randomised feasibility study testing trial methods.^9^,^11^ Finally, the third phase, a definitive, pragmatic RCT is described in this publication.

### Aims and objectives

To assess the effectiveness of the FEMuR intervention for patients after hip fracture compared with usual rehabilitation care.

Specific objectives were as follows:

#### Primary objective

To determine the effectiveness of the FEMuR intervention following surgical repair of hip fracture in older people compared with usual care, in terms of the performance of activities of daily living (ADLs) at 52 weeks’ follow-up.

#### Secondary objectives

1. To determine the effectiveness of the FEMuR intervention following surgical repair of hip fracture in older people compared with usual care, in terms of the performance of ADLs at 17 weeks’ follow-up.
2. To determine the effectiveness of the FEMuR intervention following surgical repair of hip fracture in older people compared with usual care, in terms of anxiety and depression at 17 and 52 weeks’ follow-up.
3. To assess whether the FEMuR intervention created change in fear of falling, self-efficacy, physical function, hip pain and cognitive function as potential mediators for improving ADLs at 17 and 52 weeks’ follow-up.
4. To assess whether the FEMuR intervention created change in strain, anxiety and depression in carers at 17 and 52 weeks’ follow-up.

## Methods

This was a pragmatic, multisite, parallel-group, two-armed, superiority RCT with 1:1 allocation ratio recruiting participants between August 2019 and May 2022. Outcome assessment was administered by blinded researchers; patient and carer-participants and clinicians were unblinded. A concurrent economic evaluation and an embedded process evaluation will be reported elsewhere. The trial protocol has been reported previously.^12^ Patient-participants were recruited while recovering from surgical treatment for hip fracture in 13 sites in Nottinghamshire, Norfolk, North Wales, South Wales, Kent, Derbyshire, Cheshire and Lincolnshire. They provided written informed consent to participate.

### Trial population

#### Inclusion criteria patient participants

1. Age 60 years or older.
2. Recent hip fracture (proximal femoral).
3. Surgical repair by total hip arthroplasty, hemiarthroplasty or internal fixation.
4. Living in their own home prior to hip fracture.
5. Living and receiving rehabilitation from the NHS in the area covered by the trial sites.

#### Exclusion criteria for patient participants

1. Living in residential or nursing homes prior to hip fracture.
2. Unable to understand English.
3. Lacking mental capacity to give informed consent.

#### Carer participants

We also recruited carers who were relatives or friends who provided help with ADLs on at least 4 days a week. Carer-participants provided written informed consent to complete outcome measures but did not receive any trial intervention, so did not undergo eligibility screening or randomisation.

### Randomisation

Randomisation was performed by researchers conducting baseline assessments, using secure web access to the remote randomisation site at the clinical trials unit, which generated an automated email to therapists delivering the FEMuR intervention. Randomisation was stratified by site and gender with an allocation ratio of 1:1 within each stratum. The unit of randomisation was the patient-participant.

### Study interventions

The FEMuR intervention was compared with usual rehabilitation care. This has been described in more detail previously.^8 9 12^ Usual care consisted of multidisciplinary rehabilitation delivered by the acute hospital, community hospital and community services depending on patient-participants’ needs at different times during their recovery and on local service provision. The control group received usual rehabilitation care; the intervention group received usual care plus the FEMuR intervention, which aimed to enhance usual rehabilitation by increasing self-efficacy and the amount and quality of practice, of physical exercise and ADLs.^8 12^ These were enhanced using a patient-held ‘Hip Fracture Rehabilitation Workbook’ and ‘Rehabilitation Goal Setting Diary’, provided after discharge from the acute hospital, and six additional therapy sessions in the patient participant’s home. These were delivered postdischarge, in-person, by physiotherapists or occupational therapists (including therapy assistants under their supervision) experienced in delivering rehabilitation in a community environment. In most sites, these extra sessions were delivered concurrently with usual rehabilitation, where it existed, but in one site, the additional therapy was delivered after usual care had finished. FEMuR intervention training was delivered to these therapists, regarding the use of the workbook and diary and tailoring the extra therapy sessions to patient-participants’ needs, abilities, preferences and usual rehabilitation services on offer. Therapists were asked to use their own judgement as to how the extra therapy sessions should be tailored, and in addition to setting goals and monitoring progress towards those goals, included activities such as mobility and transfers, exercises, ADLs, use of assistive devices, leisure activities, emotional well-being and family involvement. Remote meetings with the FEMuR III trial team (including the chief investigator and therapist coinvestigator examining the fidelity of the FEMuR intervention) and online forums were established to support the therapists delivering the FEMuR intervention, to answer their queries and to maintain fidelity. During the COVID-19 pandemic, the delivery of most of the extra therapy sessions, in the FEMuR intervention group, moved to remote delivery because of the lockdown restrictions.

### Outcomes

Patient-participants completed outcome measures at baseline, 17 and 52 weeks postrandomisation, administered in their homes, by a research assistant blinded to group allocation (tables1  2). The primary outcome was the Nottingham Extended Activities of Daily Living (NEADL) scale^13 14^ at 52-week follow-up. At baseline, the patient-participant was asked to recall the 4 weeks prior to hip fracture and not the 4 weeks prior to completing the questionnaire. Secondary outcomes included the NEADL at 17 weeks, the Hospital Anxiety and Depression Scale (HADS)^15^ at 17 and 52 weeks, and potential mediators of outcome at 17 and 52 weeks: Visual Analogue Scale (VAS) for hip pain intensity,^16^ Falls Efficacy Scale-International (self-efficacy)^17 18^ and VAS-Fear of Falling.^19^ EuroQol 5-Dimension 3-Level^20^ and the Client Service Receipt Inventory (CSRI)^21^ were also collected as part of a concurrent economic evaluation, which will be reported elsewhere. The research assistant assessed patient-participants’ cognitive function using the Abbreviated Mental Test Score (AMTS),^22^ grip strength^23^,^25^ at each time point, and the Short Physical Performance Battery (SPPB)^26 27^ at 17 and 52 weeks. Carer-participants completed the Caregiver Strain Index^28^ and HADS^15^ at each time point. During the COVID-19 pandemic, follow-up assessments had to be performed remotely because of the lockdown restrictions. Qualitative interviews of trial participants and therapists were collected, along with data from therapists’ records, as part of a concurrent process evaluation, which will be reported elsewhere.

**Table 1:** Baseline characteristics of patient participants in the FEMuR III trial

Baseline characteristics		Usual rehabilitation (n=100)	FEMuR intervention (n=103)
Age (years)	Mean (SD) range	80.9 (8.0) 61–100	81.2 (8.0) 60–95
Abbreviated Mental Test Score	Mean (SD) range	9.5 (0.8) 5–10	9.4 (0.9) 6–10
Gender n (%)	Female	68 (68%)	71 (69%)
Ethnicityn (%)	White (UK)	98 (98%)	101 (98%)
	American	1 (1%)	0 (0%)
	European	0 (0%)	1 (1%)
	White Canadian	0 (0%)	1 (1%)
	Missing	1 (1%)	0 (0%)
Fracture typen (%)	Intracapsular	60 (60%)	56 (54%)
	Extracapsular	31 (31%)	35 (34%)
	Missing	9 (9%)	12 (12%)
Surgery typen (%)	Total hip replacement	16 (16%)	9 (9%)
	Hemiarthroplasty	31 (31%)	44 (43%)
	Internal fixation	29 (29%)	32 (31%)
	Intramedullary nailing	20 (20%)	13 (13%)
	Missing	4 (4%)	5 (5%)
Living arrangements n (%)	Living alone	52 (52%)	49 (48%)
Place of residence before admissionn (%)	Owner occupied	87 (87%)	89 (86%)
	Housing association/local authority property	8 (8%)	8 (8%)
	Private rental	5 (5%)	5 (5%)
	Missing	0 (0%)	1 (1%)
Hospital admission length (days)	mean (SD)	8.7 (8.5)	13.6 (20.1)
Discharged directly to usual residence n (%)	Yes	66 (66%)	67 (65%)
	Missing	1 (1%)	1 (1%)
Place of discharge from acute hospitaln (%)	Owner occupied/private rental	74 (74%)	82 (80%)
	Housing association/local authority property	8 (8%)	5 (5%)
	Sheltered accommodation	0 (0%)	1 (1%)
	Residential care home	7 (7%)	5 (5%)
	Nursing home	2 (2%)	0 (0%)
	Community hospital	8 (8%)	8 (8%)
	Missing	1 (1%)	2 (2%)
Highest educational qualificationn (%)	None	24 (24%)	26 (25%)
	GCSE or equivalent	28 (28%)	21 (20%)
	A-level or equivalent	18 (18%)	21 (20%)
	Degree	8 (8%)	6 (6%)
	Higher degree	3 (3%)	5 (5%)
	Missing	19 (19%)	24 (23%)
Deprivation decile (from postcode)n (%)	1 (most deprived)	13 (13%)	4 (4%)
	2	10 (10%)	10 (10%)
	3	5 (5%)	3 (3%)
	4	7 (7%)	10 (10%)
	5	11 (11%)	11 (11%)
	6	11 (11%)	17 (17%)
	7	8 (8%)	10 (10%)
	8	8 (8%)	10 (10%)
	9	12 (12%)	9 (9%)
	10 (least deprived)	13 (3%)	14 (14%)
	Missing	2 (2%)	5 (5%)

FEMuR III, Fracture in the Elderly Multidisciplinary Rehabilitation phase III; GCSE, General Certificate of Secondary Education.

**Table 2:** Outcome measures for patient participants in the FEMuR III trial

Outcome measures (range of possible values)	Baseline		17 weeks’ follow-up		52 weeks’ follow-up		Adjusted mean difference between groups (95% CI)
	Usual rehabilitation mean (SD)	FEMuR intervention mean (SD)	Usual rehabilitation mean (SD)	FEMuR intervention mean (SD)	Usual rehabilitation mean (SD)	FEMuR intervention mean (SD)	
Primary outcome measure							
Nottingham Extended Activities of Daily Living Scale (0–66)	16.0 (5.2) n=99	16.7 (4.8)n=103	13.0 (6.1) n=83	12.9 (5.7) n=77	15.0 (5.3) n=60	13.7 (6.4) n=66	−1.9 (−3.7, 0.1)*F=3.04 p=0.0840
Secondary outcome measures							
Hospital Anxiety and Depression Scale-Anxiety (0–21)	5.7 (4.2) n=96	5.6 (4.0)n=102	4.8 (3.5)n=82	4.2 (3.6)n=77	5.1 (4.4) n=57	4.3 (3.6)n=62	0 (−1.8, 1.7)*F=0.06 p=0.8508
Hospital Anxiety and Depression Scale-Depression (0–21)	5.9 (3.6) n=96	5.6 (3.5)n=102	5.6 (3.6)n=82	4.6 (3.1)n=77	5.2 (3.7) n=57	4.8 (3.3)n=62	−0.4 (−1.8, 0.9)*F=2.55 p=0.1135
Falls Self-Efficacy International Scale (16–64)	40.3 (12.1) n=92	39.2 (13.4)n=99	35.1 (12.9) n=77	33.0 (12.3) n=74	35.0 (13.4) n=57	32.9 (13.1) n=59	0.9 (−4.7, 6.5)*F=0.06 p=0.8032
Hip Pain Intensity–Visual Analogue Scale (0–10)	5.2 (3.0) n=81	4.8 (2.7)n=84	3.0 (3.0)n=41	3.0 (3.2)n=35	4.0 (3.7) n=35	2.9 (3.8)n=34	1.1 (−2.3, 4.4)* F=0.77 p=0.3865
Fear of Falling–Visual Analogue Scale (0–10)	5.4 (3.0) n=82	5.1 (3.0)n=83	5.8 (3.4)n=44	5.4 (3.4)n=40	6.4 (3.3) n=37	5.2 (3.5)n=36	0 (−2.6, 2.6)*F=0.00 p=0.9468
Abbreviated Mental Test Score (0–10)	9.5 (0.8) n=96	9.4 (0.9)n=102	9.2 (1.7)n=81	9.3 (0.9)n=74	9.5 (0.8) n=57	9.1 (2.1)n=61	ANCOVA model not fitted
			Mann-Whitney U test p=0.6782		Mann-Whitney U test p=0.5916		
Grip strength in kg	19.8 (8.9) n=70	18.2 (7.3)n=74	16.5 (6.8) n=18	18.9 (7.9)n=19	18.4 (7.4) n=8	13.6 (8.0) n=11	−1.2 (−8.1, 5.7)*F=0.36 p=0.5722
Short Physical Performance Battery (0–12)	Not performed	Not performed	1.6 (2.8)n=41	2.9 (3.6)n=34	1.8 (3.4) n=22	2.8 (4.0)n=21	ANCOVA model not fitted
			Mann-Whitney U test p=0.0946		Mann-Whitney U test p=0.4409		

* Adjusted mean difference is for 52 week time point only, the p value takes into account all time points.

ANCOVA, analysis of covariance; FEMuR III, Fracture in the Elderly Multidisciplinary Rehabilitation phase III.

### Sample size

Based on the analysis of covariance (ANCOVA) with alpha of 5% and 90% power to detect a minimal clinically important difference on the NEADL scale of 2.4,^14^ SD=10,^29^ R^2^ of covariate=0.52) with 79% retention rate, based on the feasibility study data,^10^ 446 patient-participants needed to be recruited.

### Statistical analysis

Primary and secondary outcomes at baseline, 17 and 52 weeks follow-up were summarised for each treatment group using descriptive statistics. If outcomes were normally distributed, the difference between-group means (with 95% CIs) was reported from the repeated measures ANCOVA (accounting for 17 and 52 weeks outcomes) adjusted for baseline score and stratification factors (site and gender). Non-normally distributed outcomes were transformed and analysed as difference from baseline to ensure normality, using repeated measures ANCOVA adjusted for stratification factors. Predictors of missing data were investigated using regression models. A sensitivity analysis using a joint modelling approach was adopted to assess for any differences in longitudinal outcome between the randomised arms, adjusted for dropouts or missing values.^12^ Additional sensitivity analyses were performed excluding patient-participants who became unblinded to assessors (when the percentage of such patient-participants exceeded 5%), adjusting for those patient-participants who had any assessments during the COVID-19 pandemic, and accounting for the impact of COVID-19 restrictions on the ability for patient-participants to undertake usual ADLs. This last analysis was in relation to items in the NEADL which were not applicable during COVID-19 restrictions. This spanned seven categories such as travelling on public transport and going out socially. The remaining 15 results were summed and multiplied by 22/15, to make the shortened scale comparable to the longer NEADL scale.

The impact of engagement with the FEMuR intervention was assessed using instrumental variable regression, using the number of direct rehabilitation sessions and total time spent in direct rehabilitation sessions.^12^ Usual rehabilitation data were collected from a therapy session record completed by patient-participants and extra rehabilitation records for the FEMuR intervention completed by therapists. Missing data were supplemented by data collected by the CSRI,^21^ at the 17 and 52 weeks follow-up assessments.

Mediation analysis examined the hypothesised mechanism of change for the FEMuR intervention in terms of self-efficacy, hip pain, cognitive function, fear of falling and physical function, conditional on the overall intervention effect being significant. Each mediator was summarised using means (SD) in each treatment group and, if change from baseline was normally distributed, change from baseline ANCOVA was used to compare between the treatment groups (or if non-normal, summarised using median and IQR and analysed using Mann-Whitney U test).

### Fidelity assessment

In addition to the number of rehabilitation sessions delivered, and their mode of delivery, FEMuR intervention fidelity was assessed as part of an embedded mixed methods’ process evaluation.^12^ The results of this will be published elsewhere.

### Patient and public involvement

There has been patient and public involvement (PPI) at all stages including refining the research question, codesigning the FEMuR intervention, choosing outcomes relevant to patients, commenting on patient-facing materials, the burden of the FEMuR intervention and of trial participation. A PPI coinvestigator was an active member of the trial management group contributing to all aspects of trial conduct and dissemination.

## Results

### Trial recruitment in the COVID-19 pandemic

Recruitment to the trial was adversely affected by the COVID-19 pandemic. This led to the funder halting recruitment after 205 patient-participants had been recruited because of low recruitment rate.

### Baseline

**Figure 1:**
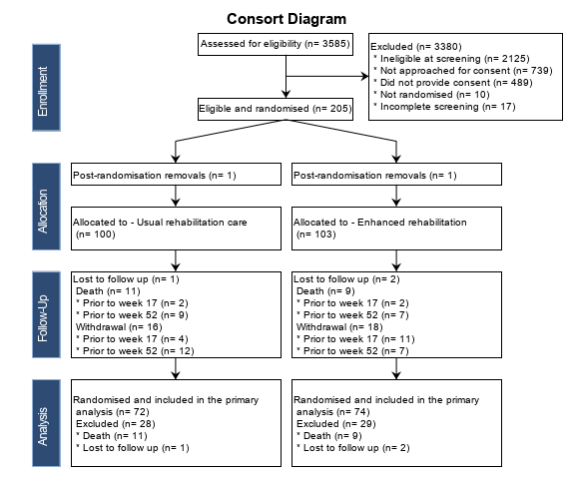
CONSORT flow diagram for the FEMuR III trial. CONSORT, Consolidated Standards of Reporting Trials; FEMuR, Fracture in the Elderly Multidisciplinary Rehabilitation phase III.

Between June 2019 and May 2022, 3585 patients with proximal femoral fracture were screened for eligibility, and 1460 (41%) were eligible (figure 1). There were 2125 ineligible, and the main reasons were not living in an area covered by the trial (823, 39%), lack of mental capacity (627, 30%) and living in residential or nursing homes prior to the fracture (447, 21%). Out of those eligible, 715 (49%) were invited to participate, 216 consented to participate and 205 (14% of the eligible population, or 29% of those invited) were randomised. The main reasons for not being approached were lack of hospital capacity (162, 22%), patients being too ill to participate (139, 19%) and discharged home (126, 17%). The main reason for declining consent was unwillingness to participate in a trial (304, 62%). From the recruited pool of patient-participants, 14 of their carers agreed to participate (7%).

The profiles of the intervention and control groups were broadly similar with respect to age, gender, ethnicity, place of residence prior to admission, educational attainment, AMTS and important comorbid conditions (table 1). The baseline scores of the outcome measures were similar between the two groups; however, grip strength was a mean of 1.6 kg greater in the control group (table 2).

### Delivery of rehabilitation sessions in the COVID-19 pandemic

The COVID-19 pandemic also affected intervention delivery (protocol amendments detailed in online supplemental table 1). Therapy records were collected for 64 patient-participants in the FEMuR intervention group. Only 28 (44%) received the planned six extra rehabilitation sessions, with three (5%) not receiving any extra sessions. The median number of extra rehabilitation sessions delivered was 4.5 (IQR: 4). The pandemic also affected the mode of delivery as most therapy sessions were switched to remote delivery by telephone. Only 17 (27%) received all six extra rehabilitation sessions in person, with no in-person contact for 21 (33%). The median number of in-person rehabilitation sessions was 2 (IQR: 6). The extra rehabilitation sessions were delivered evenly between physiotherapists and occupational therapists (50% each). The usual rehabilitation care received by both groups was very variable, with a large range and skewed distribution. The total number of rehabilitation sessions, combining usual rehabilitation sessions with the extra rehabilitation sessions in the intervention group (in-person or remote), was a median of 6 (IQR: 3) in the intervention group and 3 (IQR: 4) in the control group.

### Follow-up assessments

Two patient-participants were ineligible after randomisation (one from each group). There were 20 deaths (11 control, 9 intervention), 34 withdrew consent from further follow-up (16 control, 18 intervention) and 3 were lost to follow-up (1 control, 2 intervention) (figure 1). During COVID-19 lockdown restrictions, in-person follow-up visits were not permitted; outcomes were therefore collected remotely. Remote follow-up assessments were carried out on 108 participants at 17-week follow-up and 104 participants at 52-week follow-up, which were 67.5% and 83.2% of those included in the statistical analyses (online supplemental table 2). This meant it was not possible to perform grip strength or SPPB tests, or to record VAS assessments. One carer-participant in the intervention group withdrew consent for further follow-up for an unknown reason, and one carer-participant withdrew in the control group because their patient-participant had died.

### Primary outcome

Baseline measurements of the NEADL scale (primary outcome measure) represented patient-participants’ recall of their activity levels prior to admission following their hip fracture. Follow-up NEADL scores were lower in both groups, and neither group retained their prefracture levels of functioning. At 52 weeks, there was no significant difference between the FEMuR intervention and control groups with an adjusted mean difference of −1.9 (95% CI −3.7, −0.1). The overall repeated measures ANCOVA p value, accounting for both the 17-week and 52-week time points, was not statistically significant (p=0.084) (table 2).

### Secondary outcomes

There were no significant differences in any of the secondary outcome measures between the groups (table 2). Baseline measurement of HADS was not high, and there were no significant differences at either follow-up point. Physical function, as measured by the SPPB, was only measured in 75 (17 weeks) and 43 (52 weeks) patient-participants. Results of the SPPB were low in both groups, indicating poor balance, limited gait speed and inability to rise from a chair unaided. Outcomes were collected from only 12 carer-participants at the 17-week follow-up and eight carer-participants at 52 weeks (online supplemental table 3). The number of outcomes collected was too few to make meaningful comparisons.

### Sensitivity analyses

Adjusting for the impact of COVID-19 restrictions on patient-participants’ freedom to carry out daily activities (such as socialising and using public transport), using a change from baseline ANCOVA model, found a mean difference in NEADL of −2.0 (95% CI −3.8, –0.2) in favour of the control group by 52 weeks (p=0.07 for overall effect accounting for 17-week and 52-week measures). Adjusting according to whether patient-participants had any assessments during the COVID-19 pandemic, using a change from baseline ANCOVA, found a mean difference in NEADL of −1.9 (95% CI −3.7, –0.1) in favour of the control group by 52 weeks (p=0.09). Sensitivity analysis, excluding patient participants who became unblinded to assessors, was not required because only 4.9% of follow-up assessments were unblinded.

#### Missing data analyses

Changes from baseline ANCOVA models were used to determine predictors of NEADL score at 52 weeks, including type of surgery, age, living arrangements and comorbidity. Age was a statistically significant predictor with a co-efficient −0.1 (95% CI −0.2, 0; p=0.04) (online supplemental table 4). Logistic regression was used to determine predictors of missing outcome, but none were statistically significant (online supplemental table 5). A further sensitivity analysis, using a repeated measures ANCOVA model adjusted for age, found a mean difference in NEADL of −2.1 (95% CI −3.8, –0.3; p=0.05) in favour of the control group. Similar changes from baseline ANCOVA models were used to determine predictors of HADS anxiety and HADS depression scores at 52 weeks. Living alone was a statistically significant predictor for HADS anxiety with coefficient −1.8 (95% CI −3.4, –0.1; p=0.04). There were no statistically significant predictors of missing outcomes (determined using logistic regression). A further sensitivity analysis, using a repeated measures ANCOVA model adjusted for living alone, found no statistically significant difference with a mean difference in HADS anxiety score of −0.2 (95% CI −1.9, 1.5) in favour of the FEMuR intervention group at 52 weeks (p=0.69 accounting for both 17-week and 52- week time points).

#### Joint modelling analysis

A joint modelling analysis for the primary outcome NEADL, testing for difference in longitudinal outcome between the randomised arms accounting for dropouts and missingness, found no statistically significant difference between the groups with NEADL 0.1 (95% CI −1.1, 1.3) in favour of the FEMuR intervention group (table 3). Similar joint modelling analyses for HADS found no statistically significant difference between the groups, with HADS anxiety scale −0.5 (95% CI −1.1, 0.1) and HADS depression scale −0.4 (95% CI −0.9, 0.007) in favour of the FEMuR intervention group (table 3).

**Table 3:** Joint modelling analyses for the FEMuR III trial

Covariate	Coefficient (95% CI)	Z statistic	P value
Longitudinal (NEADL)			
Time	0.4 (0.3, 0.5)	8.1	<0.001
Treatment effect (FEMuR intervention vs usual rehabilitation care)	0.1 (−1.1, 1.3)	0.2	0.868
Survival (time in weeks to drop out)			
Treatment effect (FEMuR intervention vs usual rehabilitation care)	0.4 (−0.2, 1.0)	1.4	0.163
Overall (time in weeks) to drop out)			
Treatment effect (FEMuR intervention vs usual rehabilitation care)	0.4 (−0.1, 1.0)	1.4	0.177
Association parameter	−0.3 (−0.4, 0.2)	−6.9	<0.001
Longitudinal (HADS anxiety)			
Time	−0.1 (−0.1, 0.09)	−13.8	<0.001
Treatment effect (FEMuR intervention vs usual rehabilitation care)	−0.5 (−1.1, 0.1)	−1.6	0.118
Survival (time in weeks to drop out)			
Treatment effect (FEMuR intervention vs usual rehabilitation care)	1.3 (0.01, 2.7)	2.0	0.048
Overall (time in weeks to drop out)			
Treatment effect (FEMuR intervention vs usual rehabilitation care)	0.4 (−0.1, 0.9)	1.5	0.126
Association parameter	1.9 (1.5, 2.3)	8.6	<0.001
Longitudinal (HADS depression)			
Time*	–	–	–
Treatment effect (FEMuR intervention vs usual rehabilitation care)	−0.4 (−0.9, 0.007)	−1.9	0.05
Survival (time in weeks) to drop out)			
Treatment effect (FEMuR intervention vs usual rehabilitation care)	−0.02 (−0.5, 0.4)	−0.07	0.945
Overall (time in weeks to drop out)			
Treatment effect (FEMuR intervention vs usual rehabilitation care)	−0.04 (−0.5, 0.4)	−0.2	0.866
Association parameter	0.05 (−0.04, 0.1)	1.1	0.281

* Time of assessment has been omitted from the model due to estimation problems when it was included in the model.

FEMuR III, Fracture in the Elderly Multidisciplinary Rehabilitation phase III; HADS, Hospital Anxiety and Depression Scale; NEADL, Nottingham Extended Activities of Daily Living.

### Instrumental variable regression

The number of direct rehabilitation sessions, including usual rehabilitation in both groups and the extra rehabilitation sessions in the FEMuR intervention group, and the total time spent in direct rehabilitation sessions were used as two different potential instruments to facilitate instrumental variable analysis. The causal impact of receiving the intervention, as opposed to the intention-to-treat effect of randomised allocation provided by the repeated measures ANCOVA, was to be determined. However, randomisation was not a strong instrument, and the instrument effects were not significant in either case (p=0.62 for number of rehabilitation sessions and p=0.08 for total time spent in rehabilitation sessions). When these analyses were repeated for in-person direct sessions only, without remote telephone sessions, randomisation was not a strong instrument either (p=0.82 for number of rehabilitation sessions and p=0.18 for total time spent in rehabilitation sessions).

### Mediation analysis

Due to the non-significant overall effect of the FEMuR intervention, causal mediation analysis was not carried out (as treatment effect is a condition of mediation analysis). Most of the mediators were non-normally distributed and, therefore, summarised and analysed using non-parametric methods (online supplemental table 6).

### Unintended consequences

There were 94 serious adverse events (SAEs), including 20 deaths. In the control group, 30 patient-participants reported 53 SAEs; in the FEMuR intervention group, 33 patient-participants reported 41 SAEs. None were related to the intervention. There were 23 further falls requiring hospitalisation (11 control group, 12 intervention group), 22 fractures, including 7 hip fractures (4 control group, 3 intervention group). In addition, there were 123 non-serious AE. In the control group, 28 patient-participants reported 74 AEs; in the FEMuR intervention group, 25 patient-participants reported 49 AEs.

## Discussion

### Summary of main findings

Recruitment and retention in the trial were adversely affected by the COVID-19 pandemic. This reduced the power of the statistical analysis. At 52 weeks, there was no significant difference in NEADL score between the FEMuR intervention and control groups. There were also no significant changes in the HADS or the potential mediators (self-efficacy, hip pain intensity, fear of falling, grip strength and SPPB). Sensitivity analyses examining the impact of COVID-19 restrictions produced similar results. The median number of extra rehabilitation sessions delivered to the FEMuR intervention group was 4.5, and only a median of two were delivered in-person, which has important implications for intervention fidelity.

### Strengths and weaknesses

This definitive RCT followed previous work codeveloping the FEMuR intervention and assessing the feasibility of trial methods, according to the MRC framework for developing and evaluating complex interventions.^7^ However, this feasibility work could not foresee the impact of the COVID-19 pandemic on research project delivery, including the withdrawal of in-person visits by therapists to patient-participants’ homes. We did not manage to recruit any patient-participants from ethnic minorities, despite recruiting in areas with large ethnic minority populations. There was active PPI throughout all stages of the RCT.

We were unable to recruit the number of patient-participants indicated by our sample size calculation due to COVID-19 restrictions. The primary statistical analysis was, therefore, underpowered, with the subsequent potential for both type I and II errors. There were also 20 deaths, 34 withdrawals of consent and 3 lost to follow-up, which reduced statistical power further. We know from our previous feasibility study^10^ that compared with the total population who fracture their hip, we would recruit a younger sample with fewer complications. The patient-participants were a motivated group who were happy to participate in research projects and in rehabilitation programmes. This might have meant that those in the control group were motivated to recover their ADLs using their own resources. Lockdown restrictions imposed during the COVID-19 pandemic restricted in-person delivery of the FEMuR intervention, but we were able to continue delivering the intervention remotely. Instead of the planned six additional therapy sessions, patient-participants in the FEMuR intervention group received a median of 4.5 sessions, with only a median of two being in-person. Follow-up visits to patient-participants’ homes were also restricted. However, we were able to continue follow-up assessments remotely, but recording of VAS, measurement of grip strength and physical function assessment using the SPPB was not possible, which limited the assessment of potential mediators. We were unable to recruit many carer-participants because of visiting restrictions in hospitals during the COVID-19 pandemic. We asked participants in both groups to complete diaries of usual rehabilitation care in the community, but did not collect data on the frequency and duration of rehabilitation therapy during the hospital stay.

### Comparison with previous literature

Other studies have found that health status and quality of life improved in most patients in the first 6 months after hip fracture but did not return to prefracture levels.^30^ In contrast to the findings from this RCT, other studies have found that extended exercise rehabilitation programmes offered beyond the regular rehabilitation period improved physical functional outcomes.^31^ These programmes were more intensive than the FEMuR intervention, with home-based in-person programmes lasting up to 12 months, offering up to 56 daily home visits. A systematic review of patient perspectives of recovery after hip fracture found that full recovery was a return to prefracture activities enabling independence.^32^ Participants felt vulnerable because of anxieties about fear of falling, ability to cope at home, going out in the community and attending social events, all of which would have been made more difficult by the COVID-19 lockdown. However, our sensitivity analyses adjusting for the effect of COVID-19 restrictions on the performance of ADLs, and follow-up assessment during the pandemic, did not alter the findings. In the review, recovery was driven by a positive outlook and active engagement in the recovery process, which relied on realistic expectations and goals tailored to individual needs and activities.^32^ Finally, patient-participants were reliant on both professional and social support,^32^ which was lacking during the pandemic. Our findings did not add any support to the limited evidence for the role of self-efficacy on recovery following hip fracture.^33^

### Implications for practice, policy and research

The FEMuR intervention was not delivered as intended because of the COVID-19 pandemic lockdown restrictions. The intervention had to rely on remote methods of delivery, and the number of extra rehabilitation sessions delivered was fewer than planned. This lack of fidelity to the FEMuR intervention developed in the feasibility study^10^ may explain its lack of effectiveness in this RCT. There remains much to learn about how to deliver remote rehabilitation with fidelity, and it may not be possible to fully replace face-to-face interactions between clinicians and patients. We can speculate that in-person delivery of the planned six therapy sessions would have been more effective by facilitating an improvement in self-efficacy, the practice of ADLs and exercise, with greater professional and social support from friends and family. An embedded mixed-methods process evaluation has examined intervention delivery in more detail and will be reported elsewhere. Likewise, an economic evaluation of the FEMuR intervention will be reported elsewhere.

### Conclusions

The FEMuR intervention was not effective in improving the performance of ADLs in older people recovering from surgical repair of hip fracture compared with usual care. However, trial recruitment, delivery of the enhanced rehabilitation and follow-up were greatly affected by the COVID-19 pandemic, which may explain the lack of effectiveness.

## Supplementary material

10.1136/bmjopen-2024-091603 online supplemental file 1

## Data Availability

Data are available on reasonable request.
