# Supplementary material for: Effectiveness of a community-based rehabilitation programme following hip fracture: results from the Fracture in the Elderly Multidisciplinary Rehabilitation phase III (FEMuR III) randomised controlled trial
Source: BMJ Open. 2025 May 12;15(5):e091603. doi: 10.1136/bmjopen-2024-091603 (PMC12083281; doi:10.1136/bmjopen-2024-091603)
Supplement: online supplemental file 1 [file bmjopen-15-5-s001.docx]

**Supplemental Table 1** **Summary of protocol amendments approved by sponsor, funder and research ethics committee**

| **Version number and date** | **Summary of changes** | **Date of approval** |
| --- | --- | --- |
| V1.0 27/07/2018 | Original approved version | 31/10/2018 |
| V2.0 01/04/2019 | Inclusion of ISRCTN number, update of trial coordinator and their contact details throughout, clarification of participant trial duration, clarification of inclusion criterion, removal of stratification factor in schematic of trial design, update of visit windows for follow-up visits | 24/05/2019 |
| V3.0 04/10/2019 | Change of CTRC name to LCTC throughout, correction of typos throughout, update to confirm that patients can be recruited within the community | 28/11/2019 |
| V4.0 11/12/2019 | Amendment of time to randomisation from 4 weeks post surgery to 6 weeks post surgery, clarification of LCTC rebrand, clarification of inclusion criteria in regards to private rehab. | 03/02/2020 |
| V5.0 22/07/2020 | Update of logos throughout. Amendment was made to allow for remote follow ups and consent to protect vulnerable patients during the Covid-19 pandemic | 07/08/2020 |
| V6.0 13/11/2020 | Amendment of named Sponsor signatory throughout, removal of specific time points relating to Health Care Professional interviews | 11/03/2021 |
| V7.0 11/10/2021 | Amendment of Sponsor signatory throughout, amendment of baseline assessments to allow for consent to be taken during informed consent phone call | 08/12/2021 |

**Supplemental Table 2 Location of follow-up data collection**

| **Follow-up completion including missing data** | **Usual Rehabilitation** | **FEMuR Intervention** | **Total** |
| --- | --- | --- | --- |
| N (%) | 100 | 103 | 203 |
| **Week 17** |  |  |  |
| In person follow-up | 25 (25.0) | 27 (26.2) | 52 (25.6) |
| Follow-up data missing location | 9 (9.0) | 14 (13.6) | 23 (11.3) |
| Missing follow-up data | 8 (8.0) | 12 (11.7) | 20 (9.9) |
| Remote follow-up | 58 (58.0) | 50 (48.5) | 108 (53.2) |
| **Week 52** |  |  |  |
| In person follow-up | 9 (9.0) | 12 (11.7) | 21 (10.3) |
| Follow-up data missing location | 22 (22.0) | 23 (22.3) | 45 (22.2) |
| Missing follow-up data | 19 (19.0) | 14 (13.6) | 33 (16.3) |
| Remote follow-up | 50 (50.0) | 54 (52.4) | 104 (51.2) |
| **Follow-up completion excluding missing data** |  |  |  |
| **Week 17** |  |  |  |
| N (%) | 83 | 77 | 160 |
| In person follow-up | 25 (30.1) | 27 (35.1) | 52 (32.5) |
| Remote follow-up | 58 (69.9) | 50 (64.9) | 108 (67.5) |
| **Week 52** |  |  |  |
| N (%) | 59 | 66 | 125 |
| In person follow-up | 9 (15.3) | 12 (18.2) | 21 (16.8) |
| Remote follow-up | 50 (84.7) | 54 (81.8) | 104 (83.2) |

**Supplemental Table 3 Outcome measures for carer participants in the FEMuR III trial**

| **Outcome measures** | **Baseline** | | **17 weeks’ follow-up** | | **52 weeks’ follow-up** | |
| --- | --- | --- | --- | --- | --- | --- |
|  | **Usual rehabilitation mean (SD)** | **FEMuR Intervention mean (SD)** | **Usual rehabilitation mean (SD)** | **FEMuR Intervention mean (SD)** | **Usual rehabilitation mean (SD)** | **FEMuR Intervention mean (SD)** |
| Care-givers Strain Index (range 0-13) | 3.4 (3.2)  n=9 | 8.2 (1.9)  n=5 | 4.6 (4.1)  n=9 | 7.3 (2.9)  n=3 | 4.1 (4.1)  n=7 | 10.0  n=1 |
| Hospital Anxiety and Depression Scale - Anxiety (range 0-21) | 5.4 (3.2)  n=8 | 8.4 (2.8)  n=4 | 5.0 (3.8)  n=9 | 9.0 (1.7)  n=3 | 2.7 (2.8)  n=7 | 12.0  n=1 |
| Hospital Anxiety and Depression Scale - Depression (range 0-21) | 5.5 (3.4)  n=8 | 6.3 (1.3)  n=4 | 3.9 (3.5)  n=9 | 5.7 (0.6)  n=3 | 4.3 (3.8)  n=7 | 12.0  n=1 |

**Supplemental Table 4 Predictors of change from baseline at 52 weeks outcome data using ANCOVA models**

| **Nottingham Extended Activities of Daily Living Scale** | | | |
| --- | --- | --- | --- |
| **Predictor** |  | **Coefficient (95% CI)** | **p-value** |
| **Type of surgery** | Hemi-arthroplasty | -2.9 (-5.8, 0.0) | 0.0515 |
|  | Internal fixation | -0.3 (-3.4, 2.8) | 0.8379 |
|  | Intra-medullary nailing | -0.4 (-4.0, 3.3) | 0.8383 |
|  | Total Hip Replacement | - | - |
| **Age** |  | -0.1 (-0.2, 0.0) | 0.0417 |
| **Living arrangements** | Alone | 0.2 (-1.7, 2.0) | 0.8661 |
|  | With others | - | - |
| **Comorbidities** | No | 2.2 (-1.5, 5.9) | 0.2387 |
|  | Yes | - | - |
| **HADS Anxiety Scale** | | | |
| **Predictor** |  | **Coefficient (95% CI)** | **p-value** |
| **Type of surgery** | Hemi-arthroplasty | -0.2 (-2.9, 2.5) | 0.8909 |
|  | Internal fixation | -0.9 (-3.8, 2.0) | 0.5271 |
|  | Intra-medullary nailing | -2.0 (-5.4, 1.3) | 0.2338 |
|  | Total Hip Replacement | - | - |
| **Age** |  | -0.1 (-0.2, 0.0) | 0.1699 |
| **Living arrangements** | Alone | -1.8 (-3.4, -0.1) | 0.0366 |
|  | With others | - | - |
| **Comorbidities** | No | 2.4 (-0.9, 5.7) | 0.1561 |
|  | Yes | - | - |
| **HADS Depression Scale** | | | |
| **Predictor** |  | **Coefficient (95% CI)** | **p-value** |
| **Type of surgery** | Hemi-arthroplasty | 1.0 (-1.2, 3.1) | 0.3663 |
|  | Internal fixation | -0.4 (-2.7, 1.9) | 0.7476 |
|  | Intra-medullary nailing | 1.8 (-0.8, 4.5) | 0.1786 |
|  | Total Hip Replacement | - | - |
| **Age** |  | 0.1 (0.0, 0.2) | 0.1417 |
| **Living arrangements** | Alone | -0.3 (-1.7, 1.0) | 0.6327 |
|  | With others | - | - |
| **Comorbidities** | No | -0.4 (-3.1, 2.3) | 0.7654 |
|  | Yes | - | - |

**Supplemental Table 5 Predictors of missing change from baseline to week 52 outcome data using logistic regression models**

| **Nottingham Extended Activities of Daily Living Scale** | | | |
| --- | --- | --- | --- |
| **Predictor** |  | **Odds ratio (95% CI)** | **p-value** |
| **Type of surgery** | Hemi-arthroplasty vs Total Hip Replacement | 1.2 (0.5, 3.2) | 0.6859 |
|  | Internal fixation vs Total Hip Replacement | 1.2 (0.4, 3.2) | 0.7290 |
|  | Intra-medullary nailing vs Total Hip Replacement | 1.8 (0.6, 5.4) | 0.2845 |
| **Age** |  | 1.0 (1.0, 1.1) | 0.1713 |
| **Living arrangements** | Alone vs With others | 1.1 (0.6, 1.9) | 0.8175 |
| **Comorbidities** | No vs Yes | 1.4 (0.7, 2.9) | 0.3485 |
| **HADS Anxiety Scale** | | | |
| **Predictor** |  | **Odds ratio (95% CI)** | **p-value** |
| **Type of surgery** | Hemi-arthroplasty vs Total Hip Replacement | 1.5 (0.6, 4.0) | 0.3889 |
|  | Internal fixation vs Total Hip Replacement | 1.6 (0.6, 4.2) | 0.3672 |
|  | Intra-medullary nailing vs Total Hip Replacement | 1.8 (0.6, 5.4) | 0.2836 |
| **Age** |  | 1.0 (1.0, 1.1) | 0.2257 |
| **Living arrangements** | Alone vs With others | 1.4 (0.8, 2.4) | 0.2667 |
| **Comorbidities** | No vs Yes | 1.1 (0.6, 2.4) | 0.7044 |
| **HADS Depression Scale** | | | |
| **Predictor** |  | **Odds ratio (95% CI)** | **p-value** |
| **Type of surgery** | Hemi-arthroplasty vs Total Hip Replacement | 1.5 (0.6, 4.0) | 0.3889 |
|  | Internal fixation vs Total Hip Replacement | 1.6 (0.6, 4.2) | 0.3672 |
|  | Intra-medullary nailing vs Total Hip Replacement | 1.8 (0.6, 5.4) | 0.2836 |
| **Age** |  | 1.0 (1.0, 1.1) | 0.2257 |
| **Living arrangements** | Alone vs With others | 1.4 (0.8, 2.4) | 0.2667 |
| **Comorbidities** | No vs Yes | 1.1 (0.6, 2.4) | 0.7044 |

**Supplemental Table 6 Potential mediators of treatment effect**

| **Potential mediators** | **Follow-up period** | **Change score from baseline median (IQR)** | | **Mann Whitney** p value, |
| --- | --- | --- | --- | --- |
|  |  | **Control group** | **FEMuR intervention group** |  |
| Falls Self-Efficacy International Scale | week 17 | -6.0 (-14.0, 5.0)  n=73 | -6.5 (-18.0, 1.5)  n=72 | 0.3246 |
|  | week 52 | -1.5 (-17.0, 5.0)  n=54 | -7.0 (-17.0, 4.0)  n=57 | 0.3335 |
| Hip Pain Intensity VAS | week 17 | -2.1 (-4.7, 0.0)  n=37 | -1.8 (-5.2, -0.5) n=30 | 0.8419 |
|  | week 52 | -1.3 (-4.7, 1.9)  n=30 | -2.3 (-4.3, 0.7)  n=29 | 0.1480 |
| Fear of Falling VAS | week 17 | 0.4 (-2.2, 3.3)  n=40 | 0.3 (-1.4, 3.4)  n=34 | 0.6046 |
|  | week 52 | 0.5 (-1.7, 3.0)  n=32 | 0.2 (-2.9, 3.9)  n=30 | 0.1547 |
| Abbreviated Mental Test Score | week 17 | 0.0 (-1.0, 0.0)  n=77 | 0.0 (-1.0, 0.0)  n=73 | 0.6782 |
|  | week 52 | 0.0 (0.0, 0.0)  n=54 | 0.0 (0.0, 0.0)  n=60 | 0.5916 |
|  | | **Change score from baseline mean (SD)** | | **Student’s t-test** p value |
| Grip strength (kg) | week 17 | -0.6 (4.1)  n=17 | -0.7 (2.9)  n=18 | 0.3319 |
|  | week 52 | 0.5 (7.3)  n=7 | -4.1 (4.7)  n=11 | 0.2015 |
|  | | **Median score (IQR)** | | **Mann Whitney** p value, |
| Short Physical Performance Battery | week 17 | 0.0 (0.0, 2.0)  n=41 | 1.0 (0.0, 5.0)  n=34 | 0.0946 |
|  | week 52 | 0.0 (0.0, 2.0)  n=22 | 0.0 (0.0, 6.0)  n=21 | 0.4409 |

IQR Inter-Quartile Range; VAS Visual Analogue Scale
