# Supplementary material for: Effectiveness of a community-based rehabilitation programme following hip fracture: results from the Fracture in the Elderly Multidisciplinary Rehabilitation phase III (FEMuR III) randomised controlled trial
Source: BMJ Open. 2025 May 12;15(5):e091603. doi: 10.1136/bmjopen-2024-091603 (PMC12083281; doi:10.1136/bmjopen-2024-091603)
Supplement: Supplementary file 2 [file bmjopen-2024-091603.reviewer_comments.pdf]

PEER REVIEW HISTORY

BMJ Open publishes all reviews undertaken for accepted manuscripts. Reviewers are asked to complete a checklist review form and are provided with free text boxes to elaborate on their assessment. These free text comments are reproduced below.

ARTICLE DETAILS

Title (Provisional)

Effectiveness of a community-based rehabilitation programme following hip fracture: results from the Fracture in the Elderly Multidisciplinary Rehabilitation - phase III (FEMuR III) randomised controlled trial

Authors

Williams, Nefyn; Busse, Monica; Cooper, Rachael; Dodd, Susanna; Dorkenoo, Shanaz; Doungsong, Kodchawan; Edwards, Rhiannon Tudor; Green, Jessica; Hardwick, Ben; Lemmey, Andrew; Logan, Phillipa; Morrison, Valerie; Ralph, Penelope; Sackley, Catherine; Smith, Benjamin Edward; Smith, Toby; Spencer, Llinos Haf

---

VERSION 1 - REVIEW

Reviewer 1
Name Rupp, Markus
Affiliation University Medical Center Regensburg, Department for Trauma Surgery
Date 06-Sep-2024
COI none

The authors report on a RCT trial investigating a multidisciplinary rehab protocol which was added to standard of care. The authors do not find a difference in treatment which might be due to low numbers of recruited patients or limited patient access during the COVID 19 pandemic. The paper is well written. Rationale, methods results and discussion are excellently presented. The major limitation of statistically underpowered study is clearly described. From the reviewers point of view the authors could more emphasize in the discussion that mutlidisciplinary efforts have to underline they efficacy in daily practice since such treatment approaches are costly and time consuming. Patient selection may also be a factor which makes its application beneficial. As clinician I also recommend to highlight the aspect that telemedicine or remote treatment can not sufficiently replace interaction with physician/therapists and patients. If the authors would emphasize these aspects I would be delighted to find this manuscript published in BMJ Open.

**Reviewer**	**2**
**Name**	**Astolphi Lima, Camila**
**Affiliation**	**Universidade Cidade de São Paulo**
**Date**	**19-Sep-2024**
**COI**	**There is no competing interests.**

This study presents the results of a randomized controlled trial (FEMuR), which was significantly impacted by the pandemic. The authors have been transparent about the missing data and the protocol changes. However, I would like to better understand the content of the extra therapies delivered (such as the type of exercises and progression), as this is closely related to the effectiveness of the intervention. Additionally, I would like to know if the participants were able to use technology for the remote intervention, as this could also impact the results. Please see my comments in the attached file.

The authors were transparent in reporting the problems faced during the clinical trial. However, I would like to suggest a more in-depth analysis of the statistical methods used. The study did not include the necessary sample size and lost a considerable number of participants during the follow-up, some adjustments were required in the statistical analysis. While I believe the analysis was well conducted, I lack the expertise for a more in-depth evaluation.

I have also included comments for the authors regarding the content of the extra therapy sessions (type of exercise and progression, for example). It is unclear to me whether the exercises mentioned in the supplementary material were intended for therapy or for patients to do independently. I believe the success of the intervention is closely tied to its content. While all negative results were attributed to the pandemic, if there is any possibility of a suboptimal intervention, it should be addressed in the discussion.

BMJ Open

Manuscript ID: bmjopen-2024-091603

Thank you for the opportunity to review this manuscript. This study presents the results of a randomized controlled trial (FEMuR), which was significantly impacted by the pandemic, affecting the data collection and, consequently, the results. The authors have been transparent about the missing data and the protocol changes.

Abstract

I suggest including protocol deviations in the intervention description. For example, specify that the intervention was delivered both in person and remotely, and note that outcomes could not be collected due to restrictions. Additionally, I recommend including the number of dropouts.

Background

The introduction is well written, highlighting the significant concern regarding hip fractures and outlining the process involved in the development of FEMuR project. Here are some general comments to consider:

1. The authors use the terms "hip fracture" and "proximal femoral fracture." I suggest selecting one term and using it consistently throughout the manuscript to enhance clarity and precision. This will help avoid any confusion for readers.

2. Page 6, line 29 – "Research recommendations from the guidelines..." implies there are multiple guidelines. I assume reference number 6 pertains to this statement. Please provide the other guidelines you are referencing.

3. The NICE guidelines recommend testing the effectiveness of additional physiotherapy or occupational therapy after a hip fracture. Do you think it is important to consider the content of these therapies? Are you incorporating best practice recommendations?

Methods

1. Inclusion criteria: Please specify the timing of the fracture (e.g., acute, subacute). Although the participants were recruited from the hospital, how long after hospital discharge does the additional therapy begin? This information is important for understanding the context of the intervention.

2. Study interventions: I would like to understand what the physiotherapist and occupational therapist did during the extra therapy sessions. The TIDieR checklist indicates that materials and procedures are described on pages 9-10 and in supplementary materials 1 and 2. However, I couldn't find a detailed description of the therapy content in the protocol, and I apologize if I overlooked it. While some exercise examples are included in the workbook, it's unclear whether these exercises were used during the extra therapy sessions or were intended for patients to do independently, since section 4 states it's "to help the participants move on after the visits from the therapist." This distinction is important to understanding the actual intervention provided. I believe the success of the intervention is closely tied to its content. What are your thoughts on this?

3. Study interventions: It is not clear whether the 6 extra therapy sessions were delivered after the usual care or concurrently with it. Could you please clarify this?

4. Study interventions: In the methods section, the intervention is described as in-person; however, the results indicate that some sessions were conducted remotely. I believe the authors should address this protocol deviation in the methods section. This will create a clearer connection between the original plan and the changes made. Additionally, I recommend including a table that outlines all protocol deviations.

5. Outcomes: The same consideration regarding protocol deviations applies here. The authors should mention that assessments were not conducted in person for some participants and that not all outcomes could be collected due to pandemic restrictions.

Results

1. Baseline: The reasons for ineligibility provided in the text are not reflected in the flowchart. Are these numbers included in the "ineligible at screening" category? Clarifying this will improve the consistency between the text and the flowchart.

2. Flowchart: Please provide the reason for post-randomisation removals.

3. Flowchart: Throughout the manuscript, the authors refer to the “FEMuR rehabilitation intervention,” “intervention group,” and “enhanced rehabilitation.” I recommend selecting one term and using it consistently throughout the manuscript to enhance clarity and coherence.

4. Delivery of rehabilitation sessions: The intervention group included 103 participants, but only 64 therapy records were available. I don’t understand why data for all therapy sessions were not collected. Could you please explain this discrepancy?

5. Delivery of rehabilitation sessions: The therapy sessions were also delivered by telephone. Could you provide more details about it? The supplementary material mentions that sessions were conducted "face-to-face or remotely (via teleconference or videoconference) depending on the level of risk to the participant." It would be great to elaborate on this in the manuscript to better understand the quality of the intervention delivered. For example, were therapists able to progress the exercises during these remote sessions? Additionally, were the participants comfortable using the technology required for these sessions?

6. Delivery of rehabilitation sessions: The usual rehabilitation care was highly variable, with a wide range and skewed distribution. Could you briefly describe whether the usual care was delivered in person or remotely (if you have this information)? It’s likely that they also faced challenges due to the pandemic.

7. Follow-up assessments: The total number of in-person and remote assessments is unclear. Could you please provide the total numbers for both in-person and remote assessments?

8. Primary outcome: The subtitle is currently “Primary Outcome,” but the second paragraph discusses secondary outcomes. Please adjust the subtitle accordingly or include a separate one for the secondary outcomes to ensure clarity.

Discussion

1. The median number of extra rehabilitation sessions delivered was 4.5, which is 76.6% of the total number of sessions. What is the adherence rate of older adults in rehabilitation programs? Despite all the challenges faced, the number of sessions delivered is a notable achievement.

2. Page 21, line 18 – This is a great idea! However, it’s important to include more information about the intervention delivered in this manuscript as well. As mentioned previously, understanding the content of the intervention is crucial for interpreting its effectiveness. Providing details about the components and progression, for example, could be helpful in understanding the results.

Tables

The term “enhanced rehabilitation” is used in Table 1, while “intervention group” appears in Table 2. As I mentioned previously, the authors are not using these terms consistently. I recommend aligning the terminology throughout the entire manuscript for clarity and coherence.

**Reviewer**	**3**
**Name**	**Lim, Seung-Kyu**

Affiliation **Seoul National University Bundang Hospital, Department of Rehabilitation Medicine**

Date **23-Oct-2024**

COI **I have no confilct of interests.**

The article explores an interested topic about hip fractures, major geriatric health care problem. However, major revision is required for publication in our journal. The reviewer has provided the following comments on the article.

Despite being a well-planned and organized multicenter randomized clinical trial, it is unfortunate that more meaningful results could not be obtained due to the unexpected impact of the COVID-19 pandemic, which hindered the proper execution of this study. However, the results of this study are still significant, as they highlight how critical healthcare services, particularly those requiring in-person treatment like rehabilitation, are highly affected by severe public health crises such as infectious diseases. From this perspective, rehabilitation therapy, which is difficult to administer effectively under face-to-face restrictions, may not deliver satisfactory outcomes. Therefore, alternative strategies should be considered when such limitations arise.

Additionally, this paper lacks information about the usual rehabilitation process. It would be helpful to provide details on the total length of hospital stay post-surgery, the frequency and duration of rehabilitation therapy during hospitalization, and at what point the additional therapy sessions were initiated after discharge.

Lastly, the study flowchart suggests that a per-protocol analysis was conducted; however, in an

RCT, it is essential to present the results of an intention-to-treat analysis.

**Reviewer**	**4**
**Name**	**Gould, Daniel**
**Affiliation**	**The University of Melbourne, Department of Surgery**
**Date**	**03-Dec-2024**
**COI**	**No competing interests**

I thank the Editor for the opportunity to review this manuscript. The authors have presented an honest and balanced report of the findings of their RCT, which was unfortunately underpowered due to the implications of the COVID-19 pandemic.

I believe the reporting is transparent, and the Discussion section adequately addresses limitations and suggests reasonable interpretations of findings in light of these limitations.

I have a number of (what I believe to be) minor comments I feel should be addressed in order to bring the manuscript to the point of being suitable for publication:

- Please provide more detail on the 'joint modelling' analysis, specifically more statistical details. Presumably this was pre-specified in the trial protocol, but this could be made clearer in the manuscript with reference to the protocol to reassure readers that this was a pre-specified analysis.

- Similar to first comment, please provide more statistical details on the instrumental variable analysis, particularly regarding testing of the assumptions of this analysis such that it is valid. Again, this may have been published elsewhere by the authors, in which case a clear reference would suffice.

- How were the 'key' co-morbid conditions selected? I.e. what were the criteria by which a co-morbidity would be judged to be 'key'? Please expand upon this in the manuscript.

- General clerical point: please ensure all acronyms are defined at first use in the manuscript

- The authrs stated that "many" therapy sessions were delivered remotely by telephone. Is the exact number (and proportion of total number of therapy sessions) available for this?

- I commend the authors on adapting to the challenges posed by the COVID-19 pandemic, however the conversion to telephone therapy sessions may have influenced fidelity of the intervention. Please expand upon this potential limitation and its possible implications.

**Reviewer**	**5**
**Name**	**Kelson, Zoe**
**Affiliation**	**University of Exeter, Mathematics**

Date **20-Dec-2024**

COI **None**

This study aims to determine the effectiveness of the FEMuR intervention compared with usual NHS care.

Reviewer comments:

"The trial protocol has been reported previously [12]."

Thanks for providing a copy of the study protocol, workbook and diary, and CONSORT checklist.

"A fully documented Statistical Analysis Plan (SAP) will be written and agreed by LCTC statistical team and the IDSMC before data collection has been completed. "

Can a copy of the SAP please be provided?

"Definitive, pragmatic, multisite, parallel-group, two-armed, superiority randomised controlled trial with 1:1 allocation ratio."

The authors have designed a rigorous study.

"Patient-participants were recruited whilst recovering from surgical treatment for proximal femoral fracture in 13 sites in Nottinghamshire, Norfolk, North Wales, South Wales, Kent, Derbyshire, Cheshire and Lincolnshire. "

Can the authors please comment on whether the recruited cohort can be considered to be representative?

"Based on the analysis of covariance (ANCOVA) with alpha of 5% and 90% power to detect a minimal clinically important difference on the NEADL scale of 2.4 [27], standard deviation (SD)=10 [28], R 2 of covariate=0.52 with 79% retention rate, based on the feasibility study data [10], 446 patientparticipants would need to be recruited."

The basis for the sample size calculation is clearly communicated.

"Randomisation was stratified by site and gender with an allocation ratio of 1:1 within each stratum. The unit of randomisation was the patient participant"

and

"If outcomes were normally distributed, the difference between-group means (with 95% Confidence Intervals (CI)) were reported from the repeated measures ANCOVA (accounting for 17 and 52 week outcomes) adjusted for baseline score and stratification factors (site and gender). Non-normally distributed outcomes were transformed and analysed as difference from baseline to ensure normality, using repeated measures ANCOVA adjusted for stratification factors"

and

"The impact of engagement with the FEMuR intervention was assessed using instrumental variable regression, using the number of direct rehabilitation sessions and total time spent in direct rehabilitation sessions. "

and

"Each mediator was summarised using means (SD) in each treatment group and, if change from baseline were normally distributed, change from baseline ANCOVA was used to compare between the treatment groups (or if non-normal, summarised using median and interquartile range (IQR) and analysed using Mann-Whitney test). "

Appropriate analyses have been conducted by the authors, which suitably adjust for stratification variables.

Can the authors please clarify how they assessed normality (e.g. visual examination or a statistical test)?

"Predictors of missing data were investigated using regression models. A sensitivity analysis using a joint modelling approach was adopted to assess for any differences in longitudinal outcome between the randomised arms, adjusted for dropouts or missing values. Additional sensitivity analyses were performed excluding patient-participants who became unblinded to assessors (when the percentage of such patient-participants exceeded 5%), adjusting for those patient-participants who had any assessments during the COVID-19 pandemic, and accounting for the impact of COVID-19 restrictions on the ability for patient-participants to undertake usual ADLs."

A number of sensitivity analyses help to explore the data and robustness of the study findings.

"Missing data were supplemented by data collected by the Client Service Receipt Inventory [29], at the 17 and 52 week follow-up assessments."

Can the authors please consider conducting an additional sensitivity analysis on the approach taken for missing data (e.g. complete case analysis), or has this been achieved in the joint modelling approach which adjusts for dropouts or missing data?

"Recruitment to the trial was adversely affected by the COVID-19 pandemic. This led to the funder1 halting recruitment after 205 patient-participants had been recruited, because of low recruitment rate"

and

"This reduced the power of the statistical analysis"

Given the sample size was not reached, the study is underpowered. Can the authors please discuss this in more detail, noting the subsequent potential for both type I and II errors?

Did the authors consider exploring any subgroup analyses?

"The profiles of the intervention and control groups were broadly similar with respect to age, gender, ethnicity, place of residence prior to admission, educational attainment, AMTS and key co-morbid conditions (Table 1). The baseline scores of the outcome measures were similar between the two groups, however grip strength was a mean of 1.6 kg greater in the control group (Table 2)."

Can the authors please confirm if the change from baseline analyses adjust for this difference in baseline grip strength?

"Only 28 (44%) received the planned six extra rehabilitation sessions"

Can the authors please conduct a per protocol exploratory sensitivity analysis?

"The overall repeated measures ANCOVA p-value, accounting for both the 17- and 52-week timepoints, was only borderline significant, p=0.08) (Table 2)"

and

"using a change from baseline ANCOVA, found a mean difference in NEADL of -1.9 (95% CI -3.7, -0.1) in favour of the control group by 52 weeks (p=0.09)"

and

"using a repeated measures ANCOVA model adjusted for living alone, found a mean difference in HADS anxiety score of -0.2 (95% CI -1.9, 1.5; p=0.69) in favour of the intervention group at 52 weeks, which was not statistically significant (p=0.69 accounting for both 17 and 52 week time-points)"

and

"Similar joint modelling analyses for HADS found non-statistically significant improvements in HADS anxiety scale of -0.5 (95% CI -1.1, 0.1) and HADS depression scale of -0.4 (95% CI -0.9, 0.01) in favour of the intervention group (Table 3). "

Can the authors please re-frame the inferences, noting that the outcomes are non-significant (i.e. no statistical difference, rather than 'improvements', 'borderline' or 'in favour of')?

"removed the apparent negative effect of intervention on NEADL (0.1, 95% CI -1.1, 1.3)"

and

"a joint modelling analysis removed the apparent inferiority of the FEMuR intervention,"

In line with the above comment, no 'apparent negative effect' nor 'apparent inferiority' was found (instead, the outcomes were statistically non-significant) for this effect to be 'removed' in the sensitivity analyses. Can the authors please re-word this accordingly, in the Abstract, Results and Discussion sections?

"randomisation was not a strong instrument, and the instrument effects were not significant in either case (p=0.62 for number of rehabilitation sessions and p=0.08 for total time spent in rehabilitation sessions)"

This finding it crucial for interpreting the outcomes of this trial and for further research into the proposed intervention, as it demonstrates that the randomisation in this study was not instrumental.

"Recruitment and follow-up assessments were adversely affected by the COVID-19 pandemic. • In-person delivery of rehabilitation was not possible during COVID-19 lockdown, but rehabilitation delivery continued using remote methods. • Intervention fidelity was affected by the COVID-19 pandemic, with a reduced number of additional therapy sessions delivered to the intervention group. " [Strengths and limitations of the study]

The study limitations have been transparently disclosed, and are clearly addressed in the Discussion section.

VERSION 1 - AUTHOR RESPONSE

Reviewer 1
The paper is well written. Rationale, methods results and discussion are excellently presented. The major limitation of statistically underpowered study is clearly described.

REPLY: Thank you.

From the reviewer's point of view the authors could more emphasize in the discussion that mutlidisciplinary efforts have to underline the efficacy in daily practice since such treatment approaches are costly and time consuming. Patient selection may also be a factor which makes its application beneficial.

REPLY: Apologies, but we did not fully understand this comment. The implementation of the FEMuR intervention is discussed in more detail in the process evaluation, and cost issues are discussed in the economic evaluation, both of which have been submitted to other journals.

Highlight the aspect that telemedicine or remote treatment can not sufficiently replace interaction with physician/therapists and patients.

REPLY: The statement regarding this has been strengthened

Reviewer 2
This study presents the results of a randomized controlled trial (FEMuR), which was significantly impacted by the pandemic. The authors have been transparent about the missing data and the protocol changes.

REPLY – Thank you

I would like to better understand the content of the extra therapies delivered (such as the type of exercises and progression), as this is closely related to the effectiveness of the intervention.

REPLY – More detail about the content of the extra rehabilitation sessions has been added.

Additionally, I would like to know if the participants were able to use technology for the remote intervention, as this could also impact the results.

REPLY – Remote delivery was via telephone with the workbook and goal-setting diary providing structure. This is explored in more detail in a process evaluation paper submitted for publication in another journal.

It is unclear to me whether the exercises mentioned in the supplementary material were intended for therapy or for patients to do independently.

REPLY –The exercises shown in the Workbook shown in the supplementary materials were examples of typical exercises for participants' information. They were not prescriptive and were presented as examples of exercises that participants might perform following discussion with their therapist.

I believe the success of the intervention is closely tied to its content. While all negative results were attributed to the pandemic, if there is any possibility of a suboptimal intervention, it should be addressed in the discussion.

REPLY – We believe that the fidelity of the intervention was compromised and resulted in a suboptimal intervention.

Reviewer 3

This paper lacks information about the usual rehabilitation process. It would be helpful to provide details on the total length of hospital stay post-surgery, the frequency and duration of rehabilitation therapy during hospitalization, and at what point the additional therapy sessions were initiated after discharge.

REPLY - We have collected data on length of hospital stay, which has been added to Table 1, and is discussed more in our economic evaluation paper submitted elsewhere. We did not collect data on the frequency and duration of rehabilitation therapy during the hospital stay. This has been added as a weakness in the discussion. In most sites, the extra rehabilitation sessions were delivered concurrently with usual rehabilitation, where it existed, but in one site the additional therapy was delivered after usual care had finished.

Lastly, the study flowchart suggests that a per-protocol analysis was conducted; however, in an RCT, it is essential to present the results of an intention-to-treat analysis.

REPLY - The main analysis was by intention-to-treat.

Reviewer 4

The authors have presented an honest and balanced report of the findings of their RCT, which was unfortunately underpowered due to the implications of the COVID-19 pandemic.
I believe the reporting is transparent, and the Discussion section adequately addresses limitations and suggests reasonable interpretations of findings in light of these limitations.

REPLY: Thank you

Please provide more detail on the 'joint modelling' analysis, specifically more statistical details. Presumably this was pre-specified in the trial protocol, but this could be made clearer in the manuscript with reference to the protocol to reassure readers that this was a pre-specified analysis.

REPLY: This analysis was pre-specified with more detail in the protocol paper (reference 12 added).

Similar to first comment, please provide more statistical details on the instrumental variable analysis, particularly regarding testing of the assumptions of this analysis such that it is valid. Again, this may have been published elsewhere by the authors, in which case a clear reference would suffice.

REPLY: There is more detail about this analysis in the protocol paper (reference 12 added).

How were the 'key' co-morbid conditions selected? I.e. what were the criteria by which a co-morbidity would be judged to be 'key'? Please expand upon this in the manuscript.

REPLY: In the protocol we said that we would collect important co-morbid collections from participants' routinely collected data. The description of these has been changed to match the protocol.

General clerical point: please ensure all acronyms are defined at first use in the manuscript

REPLY:

The authors stated that "many" therapy sessions were delivered remotely by telephone. Is the exact number (and proportion of total number of therapy sessions) available for this?

REPLY: Thank you. This is discussed in the following sentence.

I commend the authors on adapting to the challenges posed by the COVID-19 pandemic, however the conversion to telephone therapy sessions may have influenced fidelity of the intervention. Please expand upon this potential limitation and its possible implications.

REPLY: These potential limitations have been discussed further in the implications section.

Reviewer: 5
Can a copy of the SAP please be provided?

REPLY: The statistical analysis plan has been uploaded.

The authors have designed a rigorous study.

REPLY: Thank you

Can the authors please comment on whether the recruited cohort can be considered to be representative?

REPLY: The recruited cohort recruited more women than men and across all socio-economic deciles. There was a geographical spread across England and Wales, but there was no ethnic diversity in the sample. The previous feasibility study included a cohort study, and recruited a younger and fitter sample.

The basis for the sample size calculation is clearly communicated.

REPLY: Thank you

Appropriate analyses have been conducted by the authors, which suitably adjust for stratification variables.

REPLY: Thank you

Can the authors please clarify how they assessed normality (e.g. visual examination or a statistical test)?

REPLY: Normality was assessed by plotting a histogram of the outcome measure scores and comparing this to a normal probability curve.

Can the authors please consider conducting an additional sensitivity analysis on the approach taken for missing data (e.g. complete case analysis), or has this been achieved in the joint modelling approach which adjusts for dropouts or missing data?

REPLY: Adjustment for missingness has been achieved in the joint modelling approach.

Given the sample size was not reached, the study is underpowered. Can the authors please discuss this in more detail, noting the subsequent potential for both type I and II errors?

REPLY: Thank you, this has been added to the discussion.

Did the authors consider exploring any subgroup analyses?

REPLY: As can be seen by the attached statistical analysis plan, we did not plan any sub-group analyses *a priori*, and we had insufficient statistical power for any *post-hoc* subgroup analyses.

"The profiles of the intervention and control groups were broadly similar with respect to age, gender, ethnicity, place of residence prior to admission, educational attainment, AMTS and key co-morbid conditions (Table 1). The baseline scores of the outcome measures were similar between the two groups, however grip strength was a mean of 1.6 kg greater in the control group (Table 2)."
Can the authors please confirm if the change from baseline analyses adjust for this difference in baseline grip strength?

REPLY: Baseline grip strength was accounted for when assessing the impact of intervention on grip strength.

"Only 28 (44%) received the planned six extra rehabilitation sessions"
Can the authors please conduct a per protocol exploratory sensitivity analysis?

REPLY: The instrumental variable regression accounts for the amount of intervention received and therefore more appropriately estimates efficacy than a per protocol analysis (which would be inherently biased due to self-selection of participants, i.e. selection bias).

Can the authors please re-frame the inferences, noting that the outcomes are non-significant (i.e. no statistical difference, rather than 'improvements', 'borderline' or 'in favour of')?

REPLY: Thank you, we agree and have re-phrased these to clearly state that there was no statistical significance.

"randomisation was not a strong instrument, and the instrument effects were not significant in either case (p=0.62 for number of rehabilitation sessions and p=0.08 for total time spent in rehabilitation sessions)"
This finding it crucial for interpreting the outcomes of this trial and for further research into the proposed intervention, as it demonstrates that the randomisation in this study was not instrumental.

REPLY: Yes, we agree.

The study limitations have been transparently disclosed, and are clearly addressed in the Discussion section.

REPLY: Thank you.

Reviewer 6

Abstract
I suggest including protocol deviations in the intervention description. For example, specify that the intervention was delivered both in person and remotely, and note that outcomes could not be collected due to restrictions. Additionally, I recommend including the number of dropouts.

REPLY – Thank you, these have been added to the abstract.

Background
1. The authors use the terms "hip fracture" and "proximal femoral fracture." I suggest selecting one term and using it consistently throughout the manuscript to enhance clarity and precision. This will help avoid any confusion for readers.

REPLY – We agree that this needs clarifying and hip fracture has been used throughout.

2. Page 6, line 29 – "Research recommendations from the guidelines…" implies there are multiple guidelines. I assume reference number 6 pertains to this statement. Please provide the other guidelines you are referencing.

REPLY – This has been amended to state 'Research recommendations from NICE guidance'

3. The NICE guidelines recommend testing the effectiveness of additional physiotherapy or occupational therapy after a hip fracture. Do you think it is important to consider the content of these therapies? Are you incorporating best practice recommendations?

REPLY – Yes indeed, best practice recommendations were included in the development of the FEMuR intervention described in detail in references 8, 9 and 12.

Methods
1. Inclusion criteria: Please specify the timing of the fracture (e.g., acute, subacute).

REPLY – Recent hip fracture stated in inclusion criteria.

Although the participants were recruited from the hospital, how long after hospital

discharge does the additional therapy begin? This information is important for understanding the context of the intervention.

REPLY – Same point as above

2. Study interventions: I would like to understand what the physiotherapist and occupational therapist did during the extra therapy sessions. The TIDieR checklist indicates that materials and procedures are described on pages 9-10 and in supplementary materials 1 and 2. However, I couldn't find a detailed description of the therapy content in the protocol, and I apologize if I overlooked it. While some exercise examples are included in the workbook, it's unclear whether these exercises were used during the extra therapy sessions or were intended for patients to do independently, since section 4 states it's "to help the participants move on after the visits from the therapist." This distinction is important to understanding the actual intervention provided. I believe the success of the intervention is closely tied to its content. What are your thoughts on this?

REPLY – As well as goal-setting and progress towards these goals, the additional therapy sessions in the FEMuR intervention included activities such as mobility and transfers, exercises, ADLs, advice on and use of assistive devices, leisure activities, emotional well-being and family involvement. The exercise examples in the workbook were illustrative and not prescriptive. The therapists decided which exercises or ADLs would be practised during the extra therapy sessions.

3. Study interventions: It is not clear whether the 6 extra therapy sessions were delivered after the usual care or concurrently with it. Could you please clarify this?

REPLY - In most sites, these extra sessions were delivered concurrently with usual rehabilitation, where it existed, but in one site the additional therapy was delivered after usual care had finished.

4. Study interventions: In the methods section, the intervention is described as in-person; however, the results indicate that some sessions were conducted remotely. I believe the authors should address this protocol deviation in the methods section. This will create a clearer connection between the original plan and the changes made.

REPLY – Thank you, this has been added.

Additionally, I recommend including a table that outlines all protocol deviations.

REPLY – This has been added as supplementary material 7.

5. Outcomes: The same consideration regarding protocol deviations applies here. The authors should mention that assessments were not conducted in person for some participants and that not all outcomes could be collected due to pandemic restrictions.

REPLY – Thank you, this has been added.

Results

1. Baseline: The reasons for ineligibility provided in the text are not reflected in the flowchart. Are these numbers included in the "ineligible at screening" category? Clarifying this will improve the consistency between the text and the flowchart.

REPLY – Thank you, this has been clarified.

2. Flowchart: Please provide the reason for post-randomisation removals.

REPLY – These were randomised in error as they did not fulfil the inclusion criteria.

3. Flowchart: Throughout the manuscript, the authors refer to the “FEMuR rehabilitation intervention,” “intervention group,” and “enhanced rehabilitation.” I recommend selecting one term and using it consistently throughout the manuscript to enhance clarity and coherence.

REPLY – Thank you, ‘FEMuR intervention’ is now used throughout the paper.

4. Delivery of rehabilitation sessions: The intervention group included 103 participants, but only 64 therapy records were available. I don’t understand why data for all therapy sessions were not collected. Could you please explain this discrepancy?

REPLY – Unfortunately, many of these were not completed despite multiple requests.

5. Delivery of rehabilitation sessions: The therapy sessions were also delivered by telephone. Could you provide more details about it? The supplementary material mentions that sessions were conducted "face-to-face or remotely (via teleconference or videoconference) depending on the level of risk to the participant." It would be great to elaborate on this in the manuscript to better understand the quality of the intervention delivered. For example, were therapists able to progress the exercises during these remote sessions? Additionally, were the participants comfortable using the technology required for these sessions?

REPLY – This is covered in the process evaluation paper. Therapists found the workbook and goal-setting diaries useful for remote interactions early on, as they provided some initial structure, but less useful as the patient-participant progressed. The emphasis shifted towards education, coaching and motivation. Physical goals became less ambitious due to concerns around managing risk and safety. Monitoring progress remotely was limited, making it difficult to ensure recovery was advancing appropriately. Several therapists felt that hybrid or telephone interventions encouraged greater self-efficacy and independence, supporting progress towards discharge. Patient-participants accepted that remote delivery was due to COVID-19 but preferred face-to-face.

6. Delivery of rehabilitation sessions: The usual rehabilitation care was highly variable, with a wide range and skewed distribution. Could you briefly describe whether the usual care was delivered in person or remotely (if you have this information)? It’s likely that they also faced challenges due to the pandemic.

REPLY – The mode of delivery of usual care was poorly recorded and we have not included it.

7. Follow-up assessments: The total number of in-person and remote assessments is unclear. Could you please provide the total numbers for both in-person and remote assessments?

REPLY – This has been added along with an explanatory table as Supplementary Material

8. Primary outcome: The subtitle is currently “Primary Outcome,” but the second

paragraph discusses secondary outcomes. Please adjust the subtitle accordingly or include a separate one for the secondary outcomes to ensure clarity.

REPLY – Thank you, the sub-heading ‘Secondary outcomes’ has been added.

Discussion

1. The median number of extra rehabilitation sessions delivered was 4.5, which is 76.6% of the total number of sessions. What is the adherence rate of older adults in rehabilitation programs? Despite all the challenges faced, the number of sessions delivered is a notable achievement.

REPLY – Thank you.

2. Page 21, line 18 – This is a great idea! However, it’s important to include more information about the intervention delivered in this manuscript as well. As mentioned previously, understanding the content of the intervention is crucial for interpreting its effectiveness. Providing details about the components and progression, for example, could be helpful in understanding the results.

REPLY – Thank you, more detail has been added. See previous comment.

Tables

The term “enhanced rehabilitation” is used in Table 1, while “intervention group” appears in Table 2. As I mentioned previously, the authors are not using these terms consistently. I recommend aligning the terminology throughout the entire manuscript for clarity and coherence.

REPLY – Relabelled FEMuR intervention.

VERSION 2 - REVIEW

**Reviewer**	**2**
**Name**	**Astolphi Lima, Camila**
**Affiliation**	**Universidade Cidade de São Paulo**
**Date**	**09-Mar-2025**
**COI**	

The authors have satisfactorily addressed all the raised questions and implemented the suggested revisions. The improvements made to the manuscript have enhanced the clarity and depth of the presented analysis. I believe it is now ready to be accepted for publication.

Reviewer 3

Name Lim, Seung-Kyu

Affiliation Seoul National University Bundang Hospital, Department of Rehabilitation Medicine

Date 13-Mar-2025

COI

Dear author

I appreciate your sincere effort in revising the manuscript. The revised version appears to be suitable for publication in this journal. Thank you for your dedication to enhancing the content.

Best regards

Reviewer 4

Name Gould, Daniel

Affiliation The University of Melbourne, Department of Surgery

Date 11-Mar-2025

COI

I thank the reviewers for their comprehensive revisions and responses to reviewer comments. I believe these have been addressed adequately.
